# Epitope Mapping by NMR of a Novel Anti-Aβ Antibody (STAB-MAb)

**DOI:** 10.1038/s41598-019-47626-2

**Published:** 2019-08-22

**Authors:** Adrián Posado-Fernández, Cláudia F. Afonso, Gonçalo Dória, Orfeu Flores, Eurico J. Cabrita

**Affiliations:** 1 UCIBIO, Faculdade de Ciências e Tecnologia, Universidade Nova de Lisboa, 2825-516 Caparica, Portugal; 2 STAB VIDA Lda., Madan Parque, Rua dos Inventores, 2825-182 Caparica, Portugal; 3 Instituto de Medicina Molecular (iMM), Avenida Professor Egas Moniz, 1649-028 Lisboa, Portugal

**Keywords:** Solution-state NMR, Neurodegeneration, Target validation

## Abstract

Alzheimer´s Disease (AD) is one of the most common neurodegenerative disorders worldwide. Excess of β-amyloid (Aβ), a peptide with a high propensity to misfold and self-aggregate, is believed to be the major contributor to the observed neuronal degeneration and cognitive decline in AD. Here, we characterize the epitope of a novel anti-Aβ monoclonal antibody, the STAB-MAb, which has previously demonstrated picomolar affinities for both monomers (K_D_ = 80 pM) and fibrils (K_D_ = 130 pM) of Aβ(1–42) and has shown therapeutic efficacy in preclinical mouse models of AD. Our findings reveal a widespread epitope that embraces several key Aβ residues that have been previously described as important in the Aβ fibrillation process. Of note, STAB-MAb exhibits a stronger affinity for the N-terminus of Aβ and stabilizes an α-helix conformation in the central to N-terminal region of the peptide, in addition to disrupting a characteristic salt-bridge of a hairpin structure present in fibrils. The NMR derived epitope supports the observed results from ThT-monitored fluorescence and electron microscopy experiments, in which STAB-MAb was shown to inhibit the formation of aggregates and promote disruption of pre-formed fibrils. In combination with the published *in vitro* and *in vivo* assays, our study highlights STAB-MAb as a rare and versatile antibody with analytical, diagnostic and therapeutic efficacy.

## Introduction

First reported in a seminal paper by Alois Alzheimer in 1907^1^, Alzheimer’s disease (AD) is today one of the most common neurodegenerative disorders, accounting for the majority of dementia cases worldwide and leading to a progressive decline in specific cognitive functions^2^. According to the 2015 World Alzheimer Report, it is also one of the most widespread disorders, affecting more than 46.8 million people worldwide and with the prevalence of AD cases projected to increase from 74.7 in 2030 to 131.5 million in 2050^3^.

One of the classical neuropathological hallmarks associated with AD is the presence of extracellular senile plaques resulting from the accumulation of β-amyloid (Aβ) peptides. These insoluble deposits of Aβ are derived from the proteolytic cleavage of a larger membrane protein, named the amyloid precursor protein (APP), following its sequential processing by β- and γ-secretases in neurons^4,5^. The second cleavage by γ-secretase can occur at different sites within the transmembrane portion of APP, resulting in the formation of Aβ fragments of different lengths that can be processed further into Aβ(1–40) and Aβ(1–42), the two main forms of Aβ^5–7^.

Aβ monomers are very susceptible to self-aggregation into different types of assemblies, including oligomers, protofibrils and fibrils^8^, which has complicated the prediction of monomeric Aβ structure and behavior under physiological conditions, as well as the understanding of the underlying fibrillation mechanisms. In addition, Aβ is mostly intrinsically unstructured and impossible to crystallize by conventional methods. Thus, the three-dimensional structure of Aβ with atomic resolution has been determined using mostly solution Nuclear Magnetic Resonance (NMR) Spectroscopy and molecular dynamic (MD) techniques. The conformation adopted by a specific Aβ fragment in solution is highly dependent on the type of conditions employed, including pH, temperature, solvent, ionic strength and concentration^9^. However, current evidence points to the formation of α-helical conformers, which is consistent with the membrane-spanning conformation of Aβ before APP cleavage, that can rapidly transition and rearrange into the characteristic β-sheet rich structures of oligomers and fibrils.

Initial studies on the solution structure of amyloid peptides have shown that in aqueous environments, Aβ(1–28) is essentially a random coil, while Aβ(1–39) adopts both random coil and β-sheet structures in equal proportions. However, in the presence of 50% trifluoroethanol (TFE), Aβ(1–28) and Aβ(1–39) adopt an α-helical conformation that transitions to an intermolecular β-sheet structure between pH 4 and 7^10^. A study of Aβ(1–40) in 40% TFE at a pH of 2.8 has elucidated the segments involved in secondary structure formation. Residues ranging from Q15 to D23 and I31 to M35 adopt an α-helix, while the remaining exist in a random coil^11^. More recently, studies performed in the absence of organic co-solvents have shown that Aβ(1–40) can diverge from random coil behavior and adopt a stable three-dimensional structure through local hydrophobic interactions between side-chains and a central helix^12^. In aqueous solutions of 50 mM NaCl at pH 7.3, Aβ(1–40) adopts a compact conformation in which residues H13 to D23 located in the central region form a 3_10_helix. The generation of this secondary structure promotes the interaction between hydrophobic residues present in the 3_10_helix and in the N- and C-termini of the peptide which stabilize the formation of a hairpin conformation with the central helix^9^.

The solution structure of Aβ(1–42) monomers has also been the subject of intense study. Initial reports had shown that 90% of the Aβ(1–42) peptide adopts a β-sheet conformation in aqueous environments, while forming α-helical structures at low and high pH values in 50% TFE. At pH values between 4 and 7, however, Aβ(1–42) transitions from its monomeric α-helix conformation and adopts an intermolecular β-sheet structure that is associated with the formation of oligomeric species^10^. A subsequent study of Aβ(1–42) in 80% hexafluoroisopropanol (HFIP), a co-solvent that promotes α-helix formation similar to TFE, has demonstrated that Aβ(1–42) displays a well-defined helical structure between residues S8 and G25. The latter is connected by a type I β-turn centered on G25 and S26 to a less regular second α-helix that spans residues K28 to G38^13^. This structural motif has also been found in Aβ(25–35)^14^. However, as the water content of this mixture is increased to approximately 80% to 90%, the Aβ(1–42) peptide sharply changes from a predominantly helical to a β-sheet structure. This transition is reversible and the previous conformation of Aβ(1–42) can be swayed by changing solvent composition. Moreover, the solved NMR structure showed that this conformational change is accompanied by the loss of the β-turn and part of the C-terminal α-helix, while the N-terminal helicity is maintained as the environment goes from apolar to essentially polar, with MD simulations revealing the formation of a possible antiparallel β-sheet comprising residues V18 to E22 and G37 to I41^15^. In these conditions, the structure of Aβ(25–35) also undergoes a significant decrease in C-terminal helicity, but maintains its β-turn structure between residues G25 and N27^14^.

Nevertheless, the two-residue discrepancy between Aβ(1–40) and Aβ(1–42) is physiologically relevant, since the dominant species in amyloid plaques is the 1–42 fragment and the Aβ(1–42): Aβ(1–40) ratio is increased in familial forms of AD^16^. Indeed, this C-terminal extension leads to considerable differences in their biophysical properties and the self-association of Aβ monomers into oligomers and fibrils is faster for Aβ(1–42) than Aβ(1–40). In this context, it is currently postulated that Aβ aggregation proceeds through a primary nucleation pathway that is dependent on nonspecific contacts between hydrophobic regions of the peptide, namely residues L17 to A21 and I31 to V40 or I31 to A42, as opposed to interactions between monomeric species with preformed β-strand conformations^17^.

In addition to monomers, the fibrillar structures of Aβ have also been extensively solved, particularly by solid-state NMR and cryo-EM. These studies have established that Aβ fibrils adopt a cross β-sheet structure, in which β-sheets are parallel and β-strands are perpendicular to the main fiber axis. In this structure, central residues form a β-strand-turn-β-strand motif while the first 10 to 17 residues remain disordered and solvent-exposed^18,19^.

The most relevant therapeutic strategy that has been pursued in the past years for AD treatment consists of passive immunotherapy^20^. In this context, several monoclonal antibodies with diverse antigen recognition mechanisms and thus targeting different species of Aβ have been developed and described. For instance, crenezumab recognizes the epitope comprising H13 through V24 and can thus inhibit Aβ aggregation by binding to the hydrophobic core (L17-A21) involved in the self-association and oligomerization of the peptide, as well as promote its disaggregation by disrupting the salt-bridge between D23 and K28 that stabilizes the hairpin structure of oligomers and fibrils^21^. This mechanism explains the high affinity of crenezumab for monomeric, oligomeric and fibrillar forms of Aβ. Solanezumab binds exclusively to Aβ monomers and oligomers, and not fibrils, by targeting the central, oligomer-nucleation region comprised by residues K16-S26 in a similar manner to crenezumab. The solved structure shows key interactions between the complementary determining regions (CDRs) of the antibody and the side-chains of residues K16, F19, F20 and D23, as well as the main-chain elements of the peptide’s backbone^22^.

Meanwhile, antibodies targeting the N-terminus of Aβ seem to be more effective in binding and clearing aggregates, which might be related to the increased exposure of these linear epitopes in plaque-deposited forms of the peptide. For example, aducanumab recognizes the epitope comprising residues E3-D7 in an extended conformation and shows a strong selectivity for fibrillar as opposed to monomeric Aβ^23^. Gantenerumab recognizes a conformational epitope composed of N-terminal (3E-V12) and central (V18-N27) residues and thus displays a high binding affinity to fibrillar and oligomeric Aβ^24,25^. Bapineuzumab, another N-terminal targeting antibody, recognizes residues D1-R5 in a helical conformation and displays a slight binding preference for plaque-deposited Aβ in regards to monomeric and oligomeric forms of the peptide^25,26^.

The STAB-MAb characterized in this study is a murine anti-Aβ monoclonal antibody that was developed with a therapeutic purpose. In a previously published study, this antibody has demonstrated picomolar affinities for both monomers (K_D_ = 80 pM) and fibrils (K_D_ = 130 pM) of Aβ(1–42) using SPR assays^27^. Immunohistochemistry and immunofluorescence staining further showed high binding affinities of STAB-MAb for diffuse, focal, and vascular amyloid deposits in mice and human samples. Western blot (WB) analysis confirmed the recognition of different monomeric and aggregated species, as well as for APP, while ELISA results showed a strong preference of the antibody for the linear regions 12–28 and 29–40, but also a weaker affinity for fragments 1–11 and 29–42 of the Aβ(1–42) peptide^28^. Moreover, the therapeutic potential of this antibody has already been tested in several pre-clinical assays, showing significant reductions in brain and circulating levels of Aβ(1–40), and particularly Aβ(1–42), in “aged” APP/PS1 transgenic mice on repeated intraperitoneal injections^28^. In a recently published work, total cognitive recovery was also confirmed in AD-like transgenic mice treated with nanoparticles functionalized with STAB-MAb^29^.

In order to contribute to the understanding of the mechanism of action of STAB-Mab, in this study we set out to identify the molecular determinants of STAB-MAb Aβ recognition. For this purpose, we have mapped the epitope of the STAB-MAb antibody by solution-state NMR spectroscopy using Aβ(1–40) and Aβ(1–42) peptides. To uncover the mechanism behind the STAB-MAb effect we have correlated the obtained NMR epitopes with the available structural data on Aβ monomers and fibrils, as well as ThT-monitored aggregation assays, TEM experiments and other published data on fibril formation. We propose a possible mechanism through which the STAB-Mab antibody inhibits and reverts the aggregation of Aβ and, therefore, precludes the formation of more toxic tertiary or quaternary structures.

## Results

### ThT-monitored fluorescence experiments indicate that STAB-MAb can inhibit Aβ(1–42) aggregation

The aggregation kinetics of Aβ in the presence of STAB-Mab were monitored by the Thioflavin T (ThT) assay. In theory, the antibody can interfere with the fibrillation pathway of Aβ by inhibiting the self-association of its monomers or, instead, by binding and disassembling pre-aggregated structures. The assays consisted in the measurement of ThT fluorescence in a monomeric solution of Aβ(1–42) for 120 minutes under conditions that facilitate or inhibit its aggregation and in the presence or absence of STAB-MAb.

**Figure 1:**
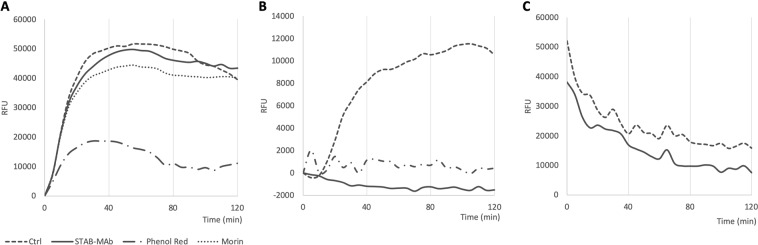
ThT fluorescence emission spectra with Aβ(1–42). RFU curves representing (**A**) a 46 µM solution of Aβ(1–42) and in presence of phenol red and morin at a 1:1 molar ratio  and STAB-Mab at a 4:1 molar ratio, (**B**) a 30 µM solution of Aβ(1–42) and in presence of phenol red and STAB-MAb at equal molar ratios, and (**C**) a 30 µM solution of pre-aggregated Aβ(1–42) and in the presence of STAB-MAb at a 1:1 molar ratio.

In a first experiment, Aβ(1–42) was resuspended in buffer at 4 °C, plated immediately at a concentration of 46 μM and incubated alone, with STAB-MAb (11.2 μM), phenol red (100 μM) and morin (100 μM) before sample reading. A significant decrease in ThT fluorescence and thus Aβ(1–42) aggregation levels was observed only in the presence of phenol red, while no differences were found following incubation with the antibody (Fig. 1A).

In a second experiment, a lower concentration of 30 μM of Aβ(1–42), in combination with a higher amount of 30 μM of STAB-MAb was used to achieve a 1:1 molar ratio between peptide and antibody. ThT curves indicated an immediate inhibition of Aβ(1–42) fibrillation, both in the presence of phenol red and STAB-MAb, with a more pronounced effect being observed in the latter case, in which fluorescence decreased approximately 2000 RFU during the first 60 minutes of experiment (Fig. 1B).

In a third and final experiment, a new Aβ(1–42) aliquot was resuspended in buffer at 4 °C and incubated for 24 hours at 37 °C to promote self-assembly. STAB-MAb was then added and aggregation monitored by ThT fluorescence. Remarkably, in this case RFU values decreased approximately 30% of their initial value in the following 2 hours, with curves for both control and antibody exhibiting a similar shape (Fig. 1C). Together, these results are a first indication that STAB-MAb can both inhibit Aβ aggregation and induce fibril disassociation.

### Electron microscopy analysis confirms that STAB-MAb can inhibit the formation of aggregates and induce fibril disassembly

**Figure 2:**
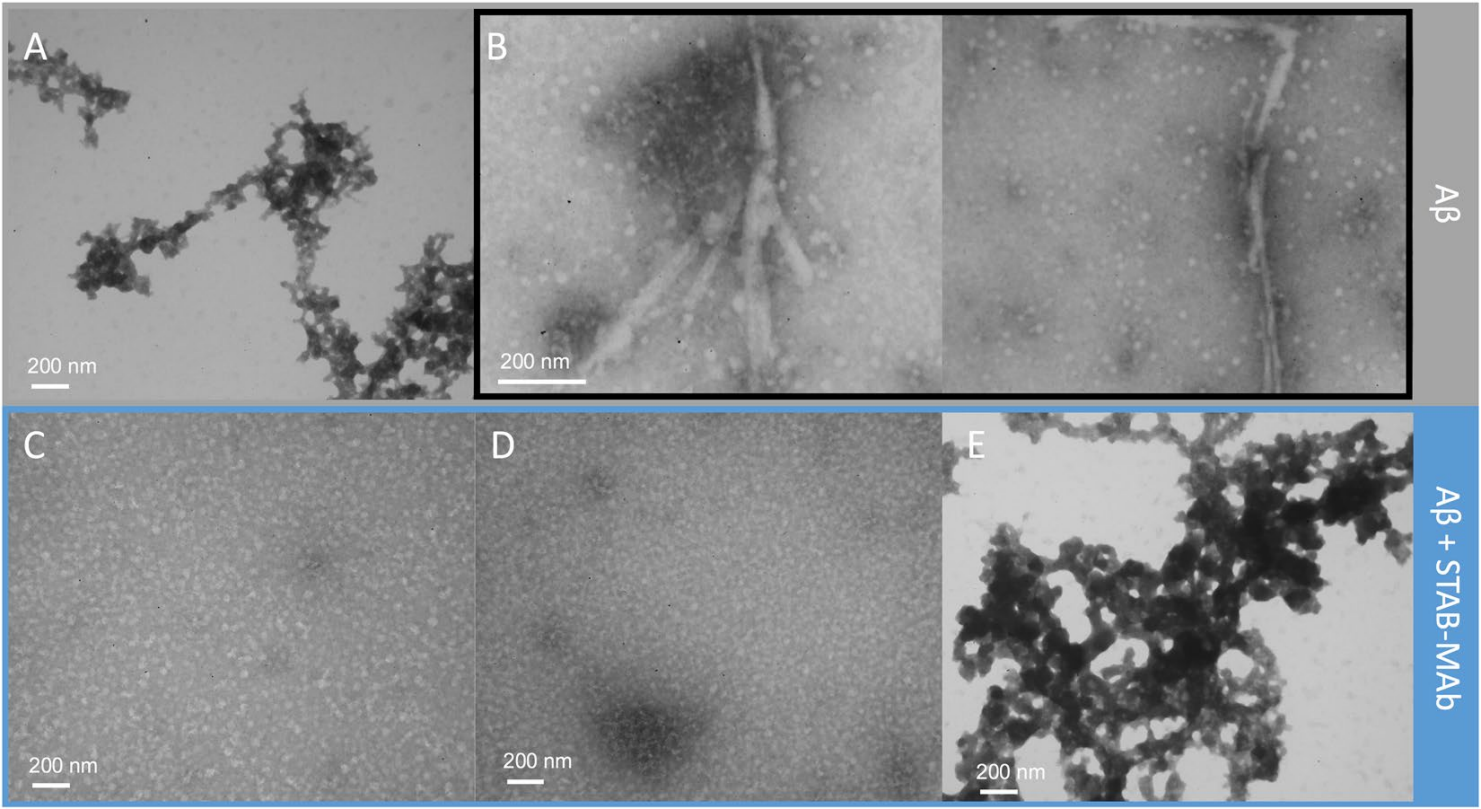
EM analysis of the effect of STAB-MAb in the formation and disassembly of Aβ(1–42) structures *in vitro*. Electron micrographs of (**A**) a freshly resuspended solution of 50 µM Aβ(1–42) in 10 mM PBS, (**B**) fibrillary assemblies in a 50 µM Aβ(1–42) solution following a 96-hour incubation period at 37 °C, (**C**) a 50 µM Aβ(1–42) solution following a 96-hour incubation period at 37 °C in presence of 25 µM STAB-MAb, (**D**) a 50 µM Aβ(1–42) solution following a 96-hour incubation period at 37 °C in presence of 50 µM STAB-MAb, and (**E**) 25 µM Aβ(1–42) solution following a 96-hour incubation period at 37 °C, succeeded by a short 24-hour incubation with 25 µM STAB-MAb at the same temperature.

Transmission electron microscopy (TEM) experiments further confirmed the ability of STAB-MAb to inhibit the *in vitro* formation of amyloid-organized aggregates and promote the disruption of pre-formed fibrils. Considering the starting point of a freshly resuspended solution of Aβ(1–42) (Fig. 2A), the peptide was able to fully assemble into organized structures such as fibrils following a 96-hour incubation period at 37 °C (Fig. 2B). However, with concomitant antibody incubation, no organized Aβ(1–42) structures were visible, but regular, small globulomers were found (Fig. 2C,D). Moreover, when the formation of Aβ(1–42) fibrillar assemblies was followed by a 24-hour incubation with STAB-MAb, no organized aggregates were observed (Fig. 2E), with micrographs unusually resembling those of the freshly resuspended peptide (Fig. 2A).

### Solution NMR experiments of Aβ(1–40) and Aβ(1–42) reveal the epitope involved in the interaction with STAB-MAb

To identify the binding epitope responsible for the interaction between amyloid and STAB-MAb, we carried out titrations of monomeric ^15^N labelled Aβ(1–40) and Aβ(1–42). These were followed by 2D ^1^H/^15^N HSQC NMR experiments using 0 to 3 molar equivalents of our antibody. The ^15^N labelled Aβ peptides were solubilized into the monomeric state as described in the Methods section.

**Figure 3:**
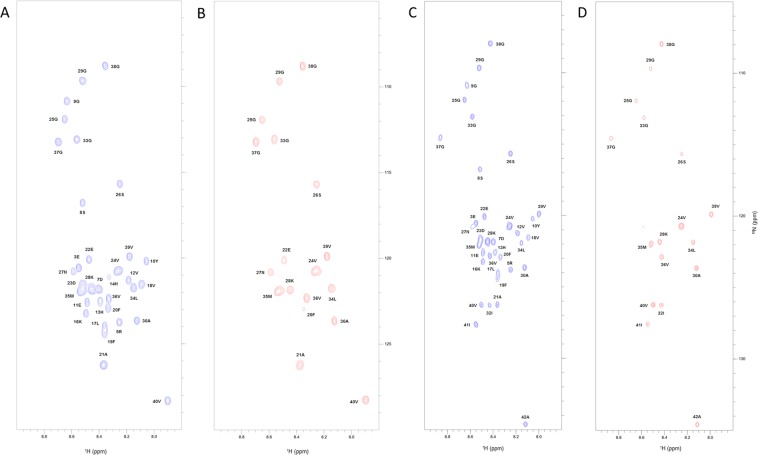
Comparison of the NMR ^1^H-^15^N SOFAST-HMQC spectrum of 20 µM Aβ(1–40) and Aβ(1–42) in the absence and presence of STAB-MAb. NMR spectra of ^15^N isotopically labelled Aβ(1–40) in the absence (**A**) and presence (**B**) of a 1:3 ratio STAB-MAb. (**D**) The same spectrum of ^15^N isotopically labelled Aβ(1–42) in the absence (**C**) and presence (**D**) of a 1:3 ratio of STAB-MAb. The figures show the region of the resonances corresponding to the backbone amide groups. Peaks were labelled according to published assignments.

The initial ^15^N-HSQC spectra of Aβ(1–40) (Fig. 3A) and Aβ(1–42) (Fig. 3C) showed sharp and well-defined peaks, which were identified according to previously published assignments^30,31^. These peaks represent the main-chain amides, indicating that samples contained the peptides in monomeric form. In the following spectra, obtained during the titration of these peptides with increasing amounts of STAB-MAb, we observed several significant chemical shift perturbations and extreme line broadening in a concentration-dependent manner, indicating that residues associated with these peaks interact directly with the antibody or are in close proximity to the binding interface.

**Figure 4:**
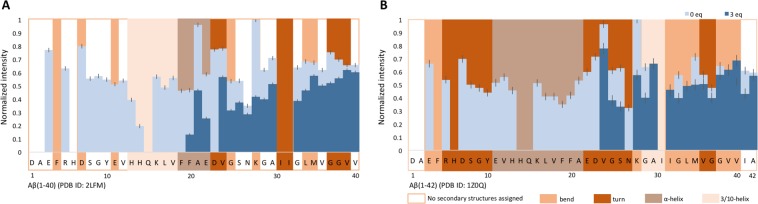
Peak intensity of Aβ(1–40) and Aβ(1–42) following titration with STAB-MAb. Histograms (**A,B**) represent the residue-specific normalized intensity (in arbitrary units) of ^15^N isotopically labeled Aβ(1–40) and Aβ(1–42) in the absence and presence of 1:3 ratio of labeled peptide to unlabeled STAB-MAb. Black bars represent the standard error (SE) determined based on the signal to noise ratio. Overlapping the histogram is a color-coded representation of the different secondary structures identified within the Aβ(1–40) peptide in 93% H_2_O/7% D2O (PDB ID. 2LFM) and for Aβ(1–42) in 70% H_2_O/30% hexafluoroisopropanol-D2O (PDB ID. 1Z0Q)^9,15^.

Indeed, combined chemical shift changes increased progressively from the N- to the C-terminal regions of the Aβ(1–40) peptide with increasing molar concentrations of STAB-MAb (Suppl. Fig. 1A). In this context, significant chemical shift perturbations were observed for residues E3, R5, V12, H13, H14, K16, L17, F20 and D23 at 0.11 molar equivalents of antibody. The first residue to exhibit extreme line broadening beyond detection was H14 with 0.33 molar equivalents of antibody. Gradually, during the two following titrations (0.66 and 1.32 molar equivalents), additional peaks displayed extreme line broadening, including E3, R5, D7, S8, G9, Y10, E11, V12, H13, K16, L17, V18 and D23 (Fig. 4A and Suppl. Fig. 2A). In agreement, we also observed a concomitant reduction in peak intensity of the entire Aβ(1–40) spectra during the first titrations (0.11 and 0.33 molar equivalents). In the following two titrations (0.66 and 1.32 molar equivalents), a group of practically consecutive residues ranging from positions E3 to V18 and D23 exhibited significant intensity decay and extreme line broadening beyond detection, while the intensity of the remaining peaks remained mostly unaltered. Interestingly, in the last titration with 3 molar equivalents of STAB-MAb, residues located in the central and C-terminal regions, ranging roughly from F20 to A40, exhibited a slight increase in peak intensity, and F19 displayed extreme line broadening (Fig. 4A and Suppl. Fig. 2A).

Similarly to the case of Aβ(1–40), combined chemical shift perturbations also increased gradually from the N- to the C-terminal regions of the Aβ(1–42) peptide with added STAB-MAb (Suppl. Fig. 1B). Significant changes were observed for R5, G9, Y10, E11, H13, K16, L17, V18, F19, D23, G29, G33 and M35 at 0.11 molar equivalents of antibody, while E3, R5 and numerous residues ranging from D7 to D23 displayed extreme line broadening beyond detection from 0.33 equivalents onwards (Fig. 4B and Suppl. Figs 1B, 2B). Of note is the disappearance of the peak associated with residue N27 at only 0.11 molar equivalents of STAB-MAb.

Following the third titration point at 0.66 equivalents, significant chemical shift changes and an increase in peak intensity were detected within the C-terminal region, namely for residues G25, S26, G29, L34, M35, V40 and I41 (Fig. 4B and Suppl. Figs 1B, 2B). Interestingly, peak intensity decreased for most residues during the first three titration points, while increasing during the last two (1.32 and 3 molar equivalents), reaching similar values to those observed in the absence of STAB-MAb (Fig. 4B).

## Discussion

The ThT fluorescence experiments allowed the *in vitro* monitoring of Aβ aggregation kinetics in the presence of our antibody. In this context, we demonstrated that STAB-MAb can completely inhibit the formation of fibril structures when present at a 1:1 molar concentration with Aβ. However, the ThT assay is not well-suited to establish whether the antibody is also capable of binding and disrupting pre-assembled Aβ aggregates. For this reason, we performed TEM analysis of several samples containing pre-formed fibrils of Aβ incubated with STAB-MAb. These experiments demonstrated that the antibody can disaggregate fibrils. In addition, we also observed that the antibody can interfere with the formation of Aβ aggregates. Together, the data provided by the ThT and TEM experiments reveals that STAB-MAb may exert its therapeutic effect through a dual mechanism, since it can both inhibit the formation of highly organized Aβ structures and also inducing the disruption of fibrils.

The NMR titrations of monomeric Aβ(1–40) and Aβ(1–42) allowed us to identify the epitope through which STAB-MAb interacts with these peptides. At low concentrations, the antibody displays a higher affinity for a subset of residues located at the N-terminal end of Aβ, as depicted by the observed chemical shift perturbations, intensity decay and extreme line broadening. With increasing antibody concentration, a few central to C-terminus residues are also affected. However, the intensity of the peaks associated with the C-terminal region recovers at higher antibody to Aβ peptide ratios.

Although with some differences, the pattern of recognition of Aβ(1–40) and Aβ(1–42) by STAB-MAb shares important similarities. The first residues to interact with the antibody are roughly located in the N-terminal region, encompassing residues E3 to D23, followed by the central to C-terminal residues, such as A30, L34, M35, V36, V39, V40 and I41. Considering our data and the structural information already available, we can propose a mechanism through which our antibody inhibits the formation of Aβ(1–40) and Aβ(1–42) aggregates. In the case of Aβ(1–40), it is conceivable that the 3_10_helix formed by residues H13 to D23 in aqueous conditions, previously described by Vivekanandan e*t al*.^9^ (PDB ID: 2LFM), is directly recognized by our antibody through an interaction with residues H13, H14, K16, L17, V18 and D23, which resulted in the intense signal decay and line broadening beyond detection of these peaks. In turn, the STAB-MAb´s interaction with this secondary structure leads to the occurrence of weaker interactions between our antibody and several C-terminal residues, which were observed as slight increases in chemical shift perturbation and decreases in intensity values. This behavior may suggest that STAB-MAb, while interacting with a number of residues within the N-terminal region of Aβ(1–40), is capable of stabilizing a pre-existing C-terminal structure of the monomeric form.

According to Tomaselli *et al*.^15^ (PDB ID: 1Z0Q), under our experimental conditions (>90% water), in contrast to the Aβ(1–40) peptide, Aβ(1–42) should have already lost most of its helical structures in favor of β conformations. This could explain the differences between our NMR data from both peptides. While the affected residues are mostly the same, the antibody shows stronger affinity for Aβ(1–42)^29^ and interacts with more residues at lower concentrations when compared to Aβ(1–40). This suggests that the antibody may have preference for the β conformations present within the Aβ(1–42) peptide, interacting with the N-terminal hydrophobic core (H13-D23), and by such, impeding the formation of structurally organized aggregates. Concurrently (and similarly to what we observed with the Aβ(1–40) peptide), with higher antibody concentrations, the C-terminal region may acquire some stable structure, as the observed increase of intensity values may suggest.

Another remarkable difference between the interaction of STAB-MAb with Aβ(1–40) and Aβ(1–42) was observed for residue N27. Its associated peak in the Aβ(1–42) spectrum displays such significant line broadening and intensity decay that it disappears at only 0.11 molar equivalents of antibody. Since it has been described that N27 is involved in the formation of a β-turn structure with G25 and S26^14^ and is essential for peptide aggregation^32^, this can represent another pathway through which STAB-MAb interferes with the fibrillation process of Aβ. The stronger interaction between N27 of Aβ(1–42) and our antibody may further explains the differential chemical shift perturbation values observed for residues G25 and S26.

As described in the introduction, residue D23 can form a salt-bridge with K28 which stabilizes a characteristic hairpin structure present in fibrils. In addition, it is hypothesized that Aβ assembly depends on interactions between hydrophobic regions, particularly L17-A21 and I31-V40 or I31-A42. Since our antibody directly interacts with both D23 and several residues within these hydrophobic regions in both Aβ(1–40) and Aβ(1–42), it is likely that this interaction precludes the β-hairpin stabilizing effect, thus inhibiting the formation of aggregates, as well as promoting fibril disruption. In turn, these binding events lead to the occurrence of weaker interactions between antibody and the I31-V40 or I31-A42 regions of the peptides, further preventing monomer assembly and destabilizing aggregates. Moreover, these hydrophobic regions, as well as the D23-K28 salt-bridge are more accessible when the Aβ peptides are in the monomeric form, which correlates with the previous observations of STAB-MAb displaying higher affinity for monomers (K_D_ = 80 pM) than fibrils (K_D_ = 130 pM)^28^.

Although the secondary structure of the two peptides in solution may be the result of differences in the conditions employed, rather than inherent differences in their structures^33,34^, in our study both Aβ(1–40) and Aβ(1–42) were analysed under the exact same experimental conditions (see Methods). We followed a protocol to overcome problems concerning the time-dependent aggregation of Aβ peptides during the NMR analysis and no concentration-dependent changes in chemical shift and line width were observed in the absence of STAB-MAb for several days, consistent with the presence of monomeric peptides. Therefore, all spectral changes observed in the presence of STAB-MAb are attributed to the interaction of the peptides with this antibody.

The NMR mapping results in combination with the already published data for STAB-MAb obtained with ELISAs^28^, indicate that its epitope is not arranged in a linear sequence as it is for other monoclonal antibodies such as aducanumab^23^. The revealed epitope of STAB-MAb encompasses several discontinuous residues, with some of these quite distant from others in the linear sequence, that can be brought together when the peptide acquires secondary or even tertiary structures in solution or when bound to the antibody. This type of epitope is likely responsible for the high affinity and broad neutralisation spectrum of STAB-MAb, as demonstrated in previous studies^27^.

In conclusion, our study highlights the unique mechanism of action of STAB-MAb, providing a structural rationale for its high affinity for Aβ monomers and fibrils. The data presented, in combination with the already published *in vitro* and *in vivo* assays, places STAB-MAb as a rare and versatile antibody for analytical, diagnostic and even therapeutic purposes^28,29,35^.

## Methods

### Production of STAB-MAb

Cells were grown at 37 °C in a humidified atmosphere containing 5% CO_2_. High glucose Glutamax DMEM (Gibco) media was supplemented with heat inactivated ultra-low IgG 10% FBS (Pan Biotech), sodium pyruvate 0.1 mM (Gibco), 10 mM HEPES (Gibco), 0.05% 2-mercaptoethanol (Gibco) and 10 mg/L gentamicin (Sigma). The supernatant was collected, filtered, stored at 4 °C and then purified with HiTrap Protein G 1 mL columns (GE Healthcare) operated through an ӒKTA system (GE Healthcare). Buffers were freshly prepared according to the manufacturer’s instructions. Following purification, the eluted fractions were dialysed at 4 °C against sterile 0.1 M PBS (Amresco) using Float-A-Lyzer cassettes (ThermoScientific). The concentration of the dialysed product was estimated by the Bradford assay using BGG (ThermoScientific) as a standard and stored at −20 °C in 1 mg/ml aliquots. When higher concentrations of STAB-MAb were required, Vivaspin 4 concentrators (Sartorius) were used.

### Thioflavin T assays

The kinetics of Aβ(1–42) aggregation were monitored using the SensoLyte® Thioflavin T β-Amyloid (1–42) Aggregation Kit (Anaspec). To measure fibril formation in 96-well black microplates, assays were performed according to the manufacturer’s instructions using morin and phenol red as the control inhibitors. The ThT fluorescence signal was monitored at 5-minute intervals for 2 hours at 37 °C using a Tecan Infinite M200. Excitation (9 nm bandwidth) and emission (20 nm bandwidth) wavelengths were 440 nm and 484 nm, respectively, while the gain was manually set at 100 and the number of flashes per read at 20.

### Transmission electron microscopy (TEM)

To evaluate the effect of STAB-MAb in the formation of amyloid aggregates, Aβ(1–42) was dissolved in DMSO at an initial concentration of 1 mM and then further diluted to a final concentration of 50 µM in PBS solutions with or without antibody. To investigate if STAB-MAb can disaggregate pre-formed assemblies, Aβ fibrils were prepared by incubating the peptide in 10 mM PBS (pH 7.4) at 37 °C for 96 hours. Samples were diluted to 5 µM in PBS, after which 30 μl were left to adsorb for 5 minutes on 300 mesh formvar-coated grids. Following a 3-minute wash with Milli-Q water, the grids were stained with 1.5% uranyl acetate. Grids were imaged using a JEOL 1200EX TEM instrument and photos were taken at x10, x25, x50, x75 and x100 K. Data was analysed with GIMP open-source software.

### NMR analysis of antibody Aβ(1–40) and Aβ(1–42) interactions

All NMR spectra were recorded on a Bruker Avance III 600 MHz spectrometer using a TCI cryo-probe. ^15^N-labelled Aβ(1–40) and Aβ(1–42) (Alexotech, Sweden) were initially dissolved into monomers as concentrated 2 mM stock solutions in DMSO-d6 (Sigma-Aldrich). Samples were then diluted 100-fold in 0.1 M PBS (Amresco) containing 5% DMSO-d6, 50 µM TSP and the STAB-MAb antibody at 1:0.1, 1:0.3, 1:0.6, 1:1.32 and 1:3 ratios at a final pH of 7.30. NMR spectra were recorded in 5 mm tubes (Norell, North Carolina) at a temperature of 278 K. 2D ^1^H/^15^N SOFAST-HMQC spectra were recorded with 2048 data points in the proton dimension and 128 data points in the nitrogen dimension with a total of 128 scans. Spectra were processed with TopSpin (Bruker, Karlsruhe) with 2048 data points in the proton and 1024 data points in the nitrogen dimension. Spectra of Aβ(1–40) and Aβ(1–42) peptides alone and in 1:0.1, 1:0.3, 1:0.6, 1:1.32 and 1:3 complexes with STAB-MAb were recorded. The ^1^H/^15^N correlated spectra were assigned according to previously published reports^30,31^. The ^15^N chemical shift was referenced indirectly to the TSP-derived ^1^H signal based on the relative gyromagnetic ratios of these nuclei. Data visualization and analysis was performed using CCPN (http://www.ccpn.ac.uk/about). Residues belonging to the binding epitopes of Aβ(1–40) and Aβ(1–42) were identified by signal intensity attenuation and combined ^1^H/^15^N chemical shift perturbations (Δδ_comb_). Residues with intensities below the noise or above the cut-off values, calculated in an iterative procedure as the corrected standard deviation to zero, were mapped to the epitope. Errors were determined based on the signal to noise ratio.

## Supplementary information

Supplementary figures
