# Supplementary material for: Epitope Mapping by NMR of a Novel Anti-Aβ Antibody (STAB-MAb)
Source: Sci Rep. 2019 Aug 22;9:12241. doi: 10.1038/s41598-019-47626-2 (PMC6706428; doi:10.1038/s41598-019-47626-2)
Supplement: Supplementary file 1 — Supplementary figures [file 41598_2019_47626_MOESM1_ESM.docx]

**Epitope Mapping by NMR of a Novel Anti-Aβ Antibody (STAB-Mab)**

Adrián Posado-Fernández^1,2^, Cláudia F. Afonso^1,3^, Gonçalo Dória^2^, Orfeu Flores^2^, Eurico J. Cabrita^1*^

^1^UCIBIO, Faculdade de Ciências e Tecnologia, Universidade Nova de Lisboa, 2825-516 Caparica, Portugal; ^2^ STAB VIDA Lda., Madan Parque, Rua dos Inventores

2825-182 Caparica, Portugal; ^3^Instituto de Medicina Molecular (iMM), Avenida Professor Egas Moniz, 1649-028 Lisboa, Portugal

*Corresponding author: Eurico J. Cabrita, UCIBIO, Faculdade de Ciências e Tecnologia, Universidade Nova de Lisboa, 2825-516 Caparica, Portugal. E-mail: [ejc@fct.unl.pt](mailto:ejc@fct.unl.pt), Phone: +351 212948358

**Supplementary Figures**


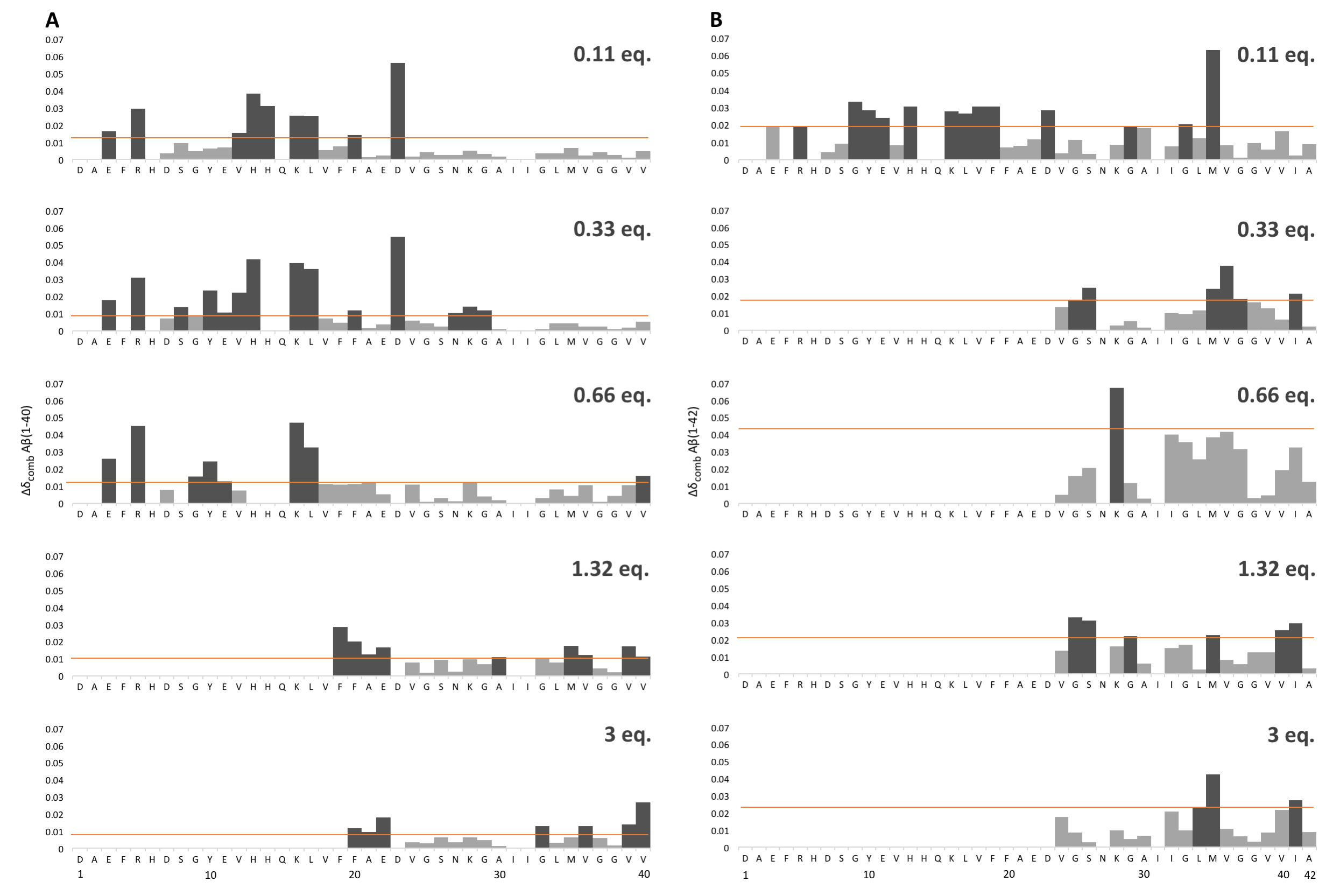


**Figure S1. Combined ^1^H/^15^N chemical shift perturbations of Aβ(1-40) and Aβ(1-42) following titration with the STAB-Mab.** Histograms represent residue-specific chemical shift changes (in ppm) of ^15^N isotopically labelled Aβ(1-40) (A) and Aβ(1-42) (B) in the presence of 1:0.11, 1:0.33, 1:0.66, 1:1.32 and 1:3 ratios of labelled peptide to unlabelled STAB-Mab, respectively. The horizontal grey line corresponds to the chemical shift cut-off value, above which residues are classed as interacting with the STAB-Mab.


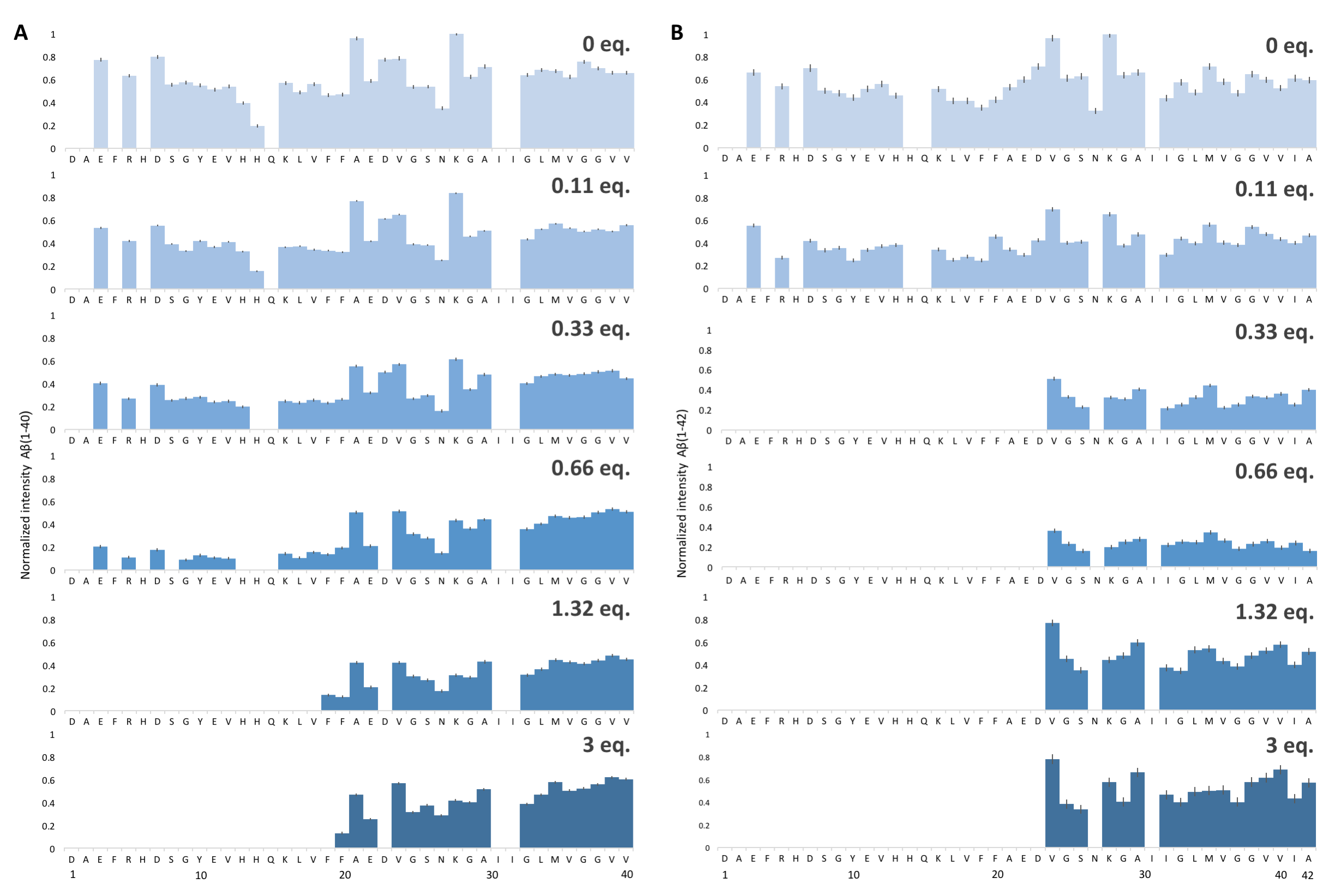


**Figure S2. Combined ^1^H/^15^N peak intensity of Aβ(1-40) and Aβ(1-42) following titration with the STAB-Mab.** Histograms represent residue-specific normalized intensity (in arbitrary units) of ^15^N isotopically labelled Aβ(1-40) (A) and Aβ(1-42) (B) in the absence and presence of 1:0.11, 1:0.33, 1:0.66, 1:1.32 and 1:3 ratios of labelled peptide to unlabelled STAB-Mab, respectively.
